# Intra-arrest-cooling CON

**DOI:** 10.1186/1471-227X-15-S1-A12

**Published:** 2015-06-24

**Authors:** Wilhelm Behringer

**Affiliations:** 1 Department for Emergency Medicine, University Hospital Jena, Germany

## 

The time of initiation of targeted temperature management in cardiac arrest patients is a matter of debate. This article will summarize the evidence for initiation of targeted temperature management already during cardiac arrest, before restoration of spontaneous circulation.

Routine care should be based on evidence of high quality. The Grading of Recommendations Assessment, Development and Evaluation (short GRADE) Working Group considers only methodologically valid human randomized clinical trials as evidence of high quality. Only two randomized clinical trials evaluated the benefit of intra-arrest cooling as compared with cooling after restoration of spontaneous circulation [1,2].

The study by Castren and colleagues investigated intra-arrest transnasal evaporative cooling within 20 minutes of the start of cardiopulmonary resuscitation (*n* = 96). Patients with in-hospital cooling after restoration of spontaneous circulation served as the control group (*n* = 104). Intra-arrest cooling was feasible and safe, but showed in comparison with in-hospital cooling no statistically significant benefit in the rate of restoration of spontaneous circulation (38% vs. 43%), survival (15% vs. 13%), or good neurological outcome (11% vs. 9%).

The study by Debaty and colleagues investigated intra-arrest cooling with infusion of up to 2 l ice-cold 0.9% saline at 100 ml/minute plus cool pads (*n* = 123), as compared with in-hospital cooling with cold saline infusion plus cooling mattress, and cold air circulation (*n* = 122). Overall, intra-arrest cooling showed no statistically significant benefit in survival as compared with in-hospital cooling (5.7% vs. 4.1%). One limitation of this study is that not all patients reached the target temperature of 32 to 34°C for 24 hours.

A pooled analysis of the two studies revealed similar results as compared with the individual studies: intra-arrest cooling versus in-hospital cooling risk ratios (95% confidence interval) were 1.6 (0.8 to 3.2) for restoration of spontaneous circulation, 1.2 (0.7 to 2.2) for survival, and 1.1 (0.6 to 1.6) for good neurological outcome.

Although pathophysiology and animal data suggest a benefit of intra-arrest hypothermia over post-arrest hypothermia, routine care should be based on evidence of high quality. Randomized clinical studies do not show benefit of intra-arrest hypothermia on neurological outcome or survival, and thus intra-arrest hypothermia should be restricted to clinical trials and not used in daily practice.
